# Percutaneous endoscopic peritoneal biopsy for a patient with unexplained ascites

**DOI:** 10.1055/a-2173-7941

**Published:** 2023-11-14

**Authors:** Li Wang, Liansong Ye, Xue Zhang, Jiamin Qin, Yong Yan, Li-Ming Wen

**Affiliations:** 1 Department of Gastroenterology, Sichuan Mianyang 404 Hospital, Mianyang, Sichuan, China; 2 Department of Gastroenterology and Hepatology, West China Hospital, Sichuan University, Chengdu, Sichuan, China; 3 Clinical Medicine College, Southwest Medical University, Luzhou, Sichuan, China

The efficacy of abdominal ultrasound, computed tomography (CT), and exfoliative cytology for determining the cause of unexplained ascites is limited. Peritoneal biopsy under laparoscopy is helpful for the diagnosis 1 . Herein, we present a novel technique of percutaneous endoscopic peritoneal biopsy (PEPB), which was performed in a 67-year-old woman with unexplained ascites.

The patient was referred to our hospital with ascites for 2 months. She had been diagnosed with tuberculous peritonitis in another hospital, but antituberculosis treatment had failed to control her ascites. She reported no other medical history. Physical examination revealed ascites, without obvious tenderness or rebound pain. Laboratory tests showed decreased albumin (37.5 g/L), and elevated ESR (72 mm/h) and CA125 (457.8 U/mL). Tests for ascites revealed it to be a transudate, and there were no tumor cells present. A computed tomography scan showed an abdominal and pelvic effusion, with blurring of the abdominal fat space ( Fig. 1 ). To determine the cause of the ascites, we performed PEPB for her in our endoscopy room ( Video 1 ).

**Fig. 1:**
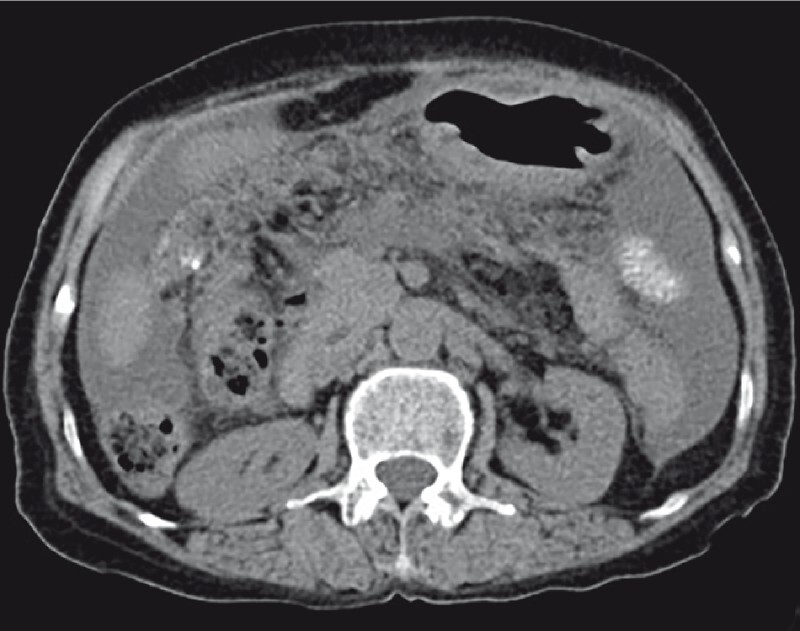
Computed tomography image showing an abdominal effusion, with blurring of the abdominal fat space.

**Video 1** Percutaneous endoscopic peritoneal biopsy is performed for a patient with unexplained ascites.


A standard upper gastrointestinal endoscope (GIF-Q260, Olympus) and laparoscopic instruments ( Fig. 2 ) were used. The whole abdominal cavity was carefully checked and yellow ascites was sucked out by the endoscope. Diffuse yellowish nodules were found in the peritoneum ( Fig. 3 ). Biopsy of the nodules was performed. Finally, the gas in the abdomen was aspirated by endoscopy, and the incision was sutured after pulling out the laparoscopic instruments. Pathology revealed serous adenocarcinoma from the ovary, confirming a diagnosis of ovarian cancer with abdominal metastasis.

**Fig. 2:**
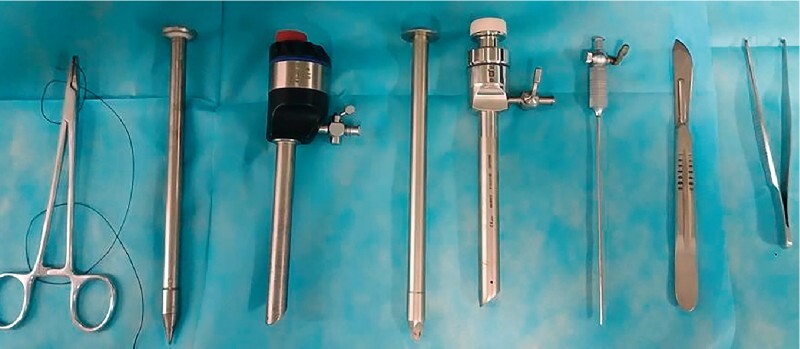
Photograph of the instruments used during percutaneous endoscopic peritoneal biopsy, including hemostatic forceps, trocars, pneumoperitoneum needle, surgical scalpel, and forceps.

**Fig. 3:**
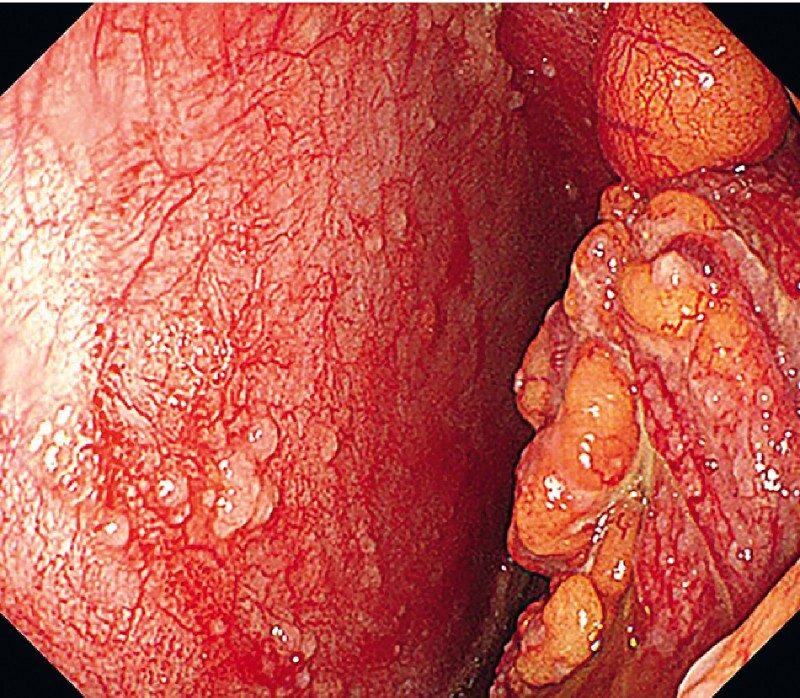
View of the peritoneum through the endoscope showing diffuse yellowish peritoneal nodules.

The patient was kept fasting for 24 hours and prophylactic antibiotics were prescribed for 3 days. She reported no obvious discomfort. Because the patient was in poor condition and could not tolerate radical surgery, chemotherapy was prescribed, after which her ascites was controlled.

Our experience demonstrates that PEPB can play a positive role in the diagnosis of unexplained ascites. Further studies are needed to assess this technique.

Endoscopy_UCTN_Code_CCL_1AG

## Notes

Endoscopy E-Videos

https://eref.thieme.de/e-videos
*E-Videos* is an open access online section of the journal *Endoscopy* , reporting on interesting cases and new techniques in gastroenterological endoscopy. All papers include a high-quality video and are published with a Creative Commons CC-BY license. Endoscopy E-Videos qualify for HINARI discounts and waivers and eligibility is automatically checked during the submission process. We grant 100% waivers to articles whose corresponding authors are based in Group A countries and 50% waivers to those who are based in Group B countries as classified by Research4Life (see: https://www.research4life.org/access/eligibility/ ). This section has its own submission website at https://mc.manuscriptcentral.com/e-videos
